# Enhancement and suppression in the visual field under perceptual load

**DOI:** 10.3389/fpsyg.2013.00275

**Published:** 2013-05-23

**Authors:** Nathan A. Parks, Diane M. Beck, Arthur F. Kramer

**Affiliations:** ^1^Beckman Institute, University of Illinois Urbana-ChampaignUrbana, IL, USA; ^2^Department of Psychological Science, University of ArkansasFayetteville, AR, USA; ^3^Department of Psychology, University of Illinois Urbana-ChampaignUrbana, IL, USA

**Keywords:** attention, perceptual load, steady-state visual evoked potential (SSVEP), N1

## Abstract

The perceptual load theory of attention proposes that the degree to which visual distractors are processed is a function of the attentional demands of a task—greater demands increase filtering of irrelevant distractors. The spatial configuration of such filtering is unknown. Here, we used steady-state visual evoked potentials (SSVEPs) in conjunction with time-domain event-related potentials (ERPs) to investigate the distribution of load-induced distractor suppression and task-relevant enhancement in the visual field. Electroencephalogram (EEG) was recorded while subjects performed a foveal go/no-go task that varied in perceptual load. Load-dependent distractor suppression was assessed by presenting a contrast reversing ring at one of three eccentricities (2, 6, or 11°) during performance of the go/no-go task. Rings contrast reversed at 8.3 Hz, allowing load-dependent changes in distractor processing to be tracked in the frequency-domain. ERPs were calculated to the onset of stimuli in the load task to examine load-dependent modulation of task-relevant processing. Results showed that the amplitude of the distractor SSVEP (8.3 Hz) was attenuated under high perceptual load (relative to low load) at the most proximal (2°) eccentricity but not at more eccentric locations (6 or 11°). Task-relevant ERPs revealed a significant increase in N1 amplitude under high load. These results are consistent with a center-surround configuration of load-induced enhancement and suppression in the visual field.

Under natural viewing conditions, the visual field is cluttered with a multitude of salient yet irrelevant stimuli. Only a subset of these stimuli are relevant for a given behavior or goal. As such, the visual system constantly performs the complex process of selecting behaviorally relevant stimuli whilst ignoring extraneous stimuli. This process of enhancing apposite stimuli and suppressing irrelevant stimuli is known as selective attention. Studies of selective visual attention have shown that when attention (not necessarily gaze) is directed to a peripheral spatial location, manual responses occur more rapidly (Posner, 1980; Posner et al., 1980) and attended stimuli are perceived as more vibrant (Prinzmetal et al., 1997, 1998; Carrasco et al., 2004; Liu et al., 2009), effects attributable to an enhanced neural response within extrastriate visual cortex (Kastner et al., 1998; Martinez et al., 1999; Silver et al., 2007). In addition to the enhanced visual responses at attended regions, neural representations of space adjacent to an attended location are inhibited, indicating that attention takes on a center-surround configuration, enhancing attended space and suppressing the surrounding area (Müller and Kleinschmidt, 2004; Müller et al., 2005; Hopf et al., 2006; Boehler et al., 2009).

Attentional enhancement and suppression of stimuli in the visual field does not occur invariably but is modulated by the demands of the task at hand. Behavioral studies have shown that distractors cause less interference when the attentional demands (perceptual load) of a task increase (Lavie and Tsal, 1994; Lavie, 1995). The perceptual load theory of attention proposes that such distractor filtering occurs because available attentional resources are diverted from distractor processing and allotted to performance of an attentionally demanding task (Lavie and Tsal, 1994; Lavie, 1995). Neurophysiological studies have supported the theory's general propositions, demonstrating that high perceptual load attenuates visual cortical responses to extraneous distractor stimuli (Kramer et al., 1988; Rees et al., 1997; Handy and Mangun, 2000; Handy et al., 2001; Berman and Colby, 2002; Pinsk et al., 2004; Schwartz et al., 2005; Rorden et al., 2008; Rauss et al., 2009, 2012; Parks et al., 2011). Despite these neurophysiological studies, perceptual load theory has not specified the neural substrate that underlies the induction of distractor filtering.

Torralbo and Beck (2008) have described a candidate neural mechanism of perceptual load, proposing that load-induced distractor filtering is a consequence of a top–down biasing signal initiated by the need to resolve neural competition between local representations in visual cortices. These competitive interactions have also been referred to as surround suppression; that is, stimuli are not processed independently but are influenced (suppressed) by surrounding stimuli (Blakemore and Tobin, 1972; Snowden et al., 1991; Knierim and Van Essen, 1992; Miller et al., 1993; Kastner et al., 1998, 2001; Reynolds et al., 1999; Bair et al., 2003). In keeping with such suppressive interactions, the presence of nearby stimuli can impair performance on a variety of tasks (Cave and Zimmerman, 1997; Bahcall and Kowler, 1999; Mounts, 2000; Kristjánsson et al., 2002; McCarley et al., 2004; Alvarez and Franconeri, 2007; Shim et al., 2008; Hilimire et al., 2009; Franconeri et al., 2010; Chan and Hayward, 2012). Top–down attention, however, serves to isolate the attended items from their surround (Moran and Desimone, 1985; Luck et al., 1997; Recanzone et al., 1997; Kastner et al., 1998; Reynolds et al., 1999; Recanzone and Wurtz, 2000; Sundberg et al., 2009) that is, the influence of the unattended stimuli is suppressed. According to biased competition theory (Reynolds et al., 1999) and normalization models of attention (Reynolds and Heeger, 2009), the suppression of unattended stimuli is a natural consequence of the inherent inhibitory interactions in visual cortex. By enhancing a target, competitively connected surrounding stimuli will necessarily be suppressed. Such models make two predictions. First, the degree of target enhancement will determine the degree of distractor suppression; in other words, if increasing the load of a task results in a need for greater enhancement of the target this should be accompanied by, as perceptual load theory predicts, greater suppression of unattended stimuli. Second, if local competitive interactions underlie load-dependent suppression then suppression should be greatest for distractor locations that are more likely to share local inhibitory interactions with the attended stimulus; that is, distractor locations most proximal to the attended target should be suppressed more than those that are more distant.

Here, we examined the spatial distribution of load-dependent enhancement and distractor suppression by parametrically manipulating distractor eccentricity during performance of a foveal visual discrimination task that varied between low and high perceptual load. Frequency-domain steady-state visual evoked potentials (SSVEPs) were measured in response to peripheral distractor stimuli and were used to evaluate distractor suppression (Müller et al., 1998a,b; Müller and Hübner, 2002; Müller and Kleinschmidt, 2003; Keitel et al., 2010; Parks et al., 2011). Time-domain event-related potentials (ERPs) were also recorded in response to task-relevant foveal stimuli and were used to evaluate attentional modulation of foveal visual processing. In accordance with Torralbo and Beck (2008) and the predictions of biased competition and normalization theories, we predicted that foveal perceptual load should result in an enhanced visual response to foveal stimuli but that this enhancement should also be associated with increased distractor suppression (filtering). Furthermore, as predicted by biased competition and normalization theory, this increased suppressive drive should be strongest at spatial locations most proximal to the attentionally demanding stimulus (i.e., foveal target). As such, we predicted that foveal load should produce the strongest suppression at the most proximal distractor locations.

## Method

### Subjects

Twenty subjects (11 females, mean age = 21.5 years) were recruited from the University of Illinois Urbana-Champaign. All subjects reported normal or corrected to normal vision. Written informed consent was obtained prior to experimentation. All procedures were approved by the University of Illinois Institutional Review Board. Subjects were paid $10 per hour for their participation in the experiment.

### Stimuli and procedure

**Figure 1:**
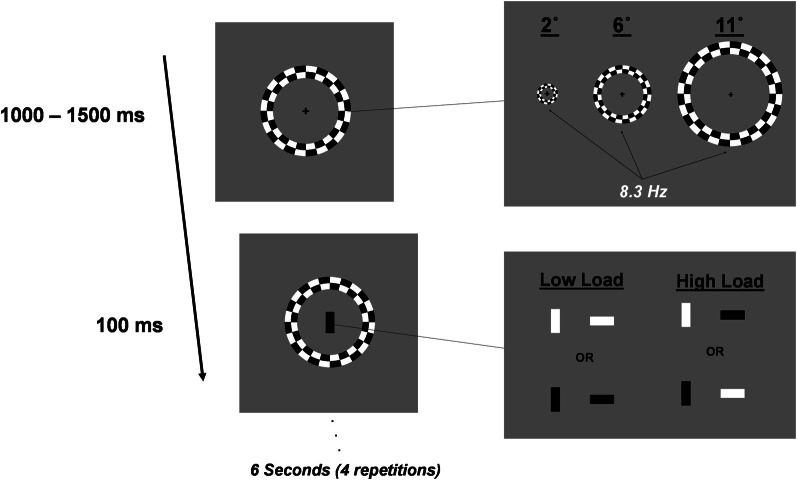
**Schematic representation of stimuli and trial progression.** Distractor stimuli were one of three rings centered at 2, 6, or 11° from fixation. Distractor stimuli contrast reversed at 8.3 Hz. Foveal stimuli were black or white rectangles oriented vertically or horizontally. Subjects performed a go/no-go task, responding to two targets that were assigned for a block of trials. Trials length was 6.0 s. Distractors contrast-reversed for the duration of the trial. Task stimuli were presented randomly every 1000–1500 ms. Four task stimuli occurred during each 6.0 s trial and were randomly selected (with replacement) from the four possible go/no-go task stimuli.

Subjects performed a foveal go/no-go task in the presence of irrelevant distractor rings that contrast reversed at a rate of 8.3 Hz. Trials were 6.0 s in duration. Every 1000–1500 ms of this trial one of four rectangles was flashed at fixation for 100 ms. Rectangles (1.0 × 0.5°) were black or white and oriented either horizontally or vertically. Four such task stimuli were randomly selected and presented during a trial. For a block of trials, two of the four rectangles were assigned as targets. Perceptual load was manipulated between blocks. In Low Load blocks, targets were assigned such that they could be discriminated from non-targets by color alone whereas high load blocks required discrimination of a conjunction of color and orientation (Figure 1). For example, in a low load block, targets may be assigned as vertical white and horizontal white rectangles whereas in a high load block targets may be vertical white and horizontal black. High load blocks were expected to be much more attentionally demanding than low load blocks (Treisman and Gelade, 1980). Subjects were instructed to respond to target rectangles as quickly as possible and withhold responses to non-target rectangles. This go/no-go task was performed in isolation or in the context of peripheral checkerboard rings. Peripheral ring stimuli consisted of one of three rings positioned at eccentricities of 2, 6, or 11° from fixation. Ring size was scaled for cortical magnification according to the method described in Carrasco and Frieder (1997). Rings contrast reversed at 8.3 Hz for the entirety of the 6.0 s trial. Peripheral rings were irrelevant to the central go/no-go task and subjects were instructed to ignore them.

Subjects completed four practice blocks of four trials followed by eight blocks of 40 trials, each 6 s in duration. The order of perceptual load conditions was counterbalanced between subjects. Within a block there was an equal weighting of peripheral distractor trial types (2, 6, 11°, and no-distractor). Time-domain ERPs were measured in response to go/no-go task stimuli and frequency-domain SSVEPs were measured in response to peripheral contrast-reversing rings.

### EEG recording and analysis

Electroencephalogram (EEG) was recorded from 30 Ag-AgCl scalp electrodes with a Synamps 2 amplifier (Neuroscan, El Paso, TX). Electrodes were positioned according to the modified 10–20 system at the following locations: O1, Oz, O2, P7, P3, Pz, P4, P8, TP7, CP3, CPz, CP4, TP8, T7, C3, Cz, C4, T8, FT7, FC3, FCz, FC4, FT8, F7, F3, Fz, F4, F8. Vertical electrooculogram (VEOG) and horizontal electrooculogram (HEOG) were formed from bipolar channels calculated from electrodes positioned above and below the left eye and on the outer canthi of the left and right eye. EEG was referenced to right mastoid, sampled at 1000 Hz, and band-pass filtered from 0.1 to 40 Hz. Offline, data were re-referenced to the average of the two mastoid channels. Electrodes were selected for analysis based on previous literature and grand average scalp distributions across all conditions. ANOVAs with more than 2° in the numerator were corrected for sphericity using a Huynh-Feldt correction. An alpha level of 0.05 was used as the criteria for significance for all statistical tests.

#### SSVEPs

For steady-state data, EEG was epoched into segments of 4096 ms, beginning 1500 ms into the trial. This time window was chosen as visual responses to contrast reversing stimuli would have achieved a steady-state, it ensured the presentation of a central task-relevant stimulus, and provided a power of two number of data points (4096) necessary for the fast Fourier transform (FFT) algorithm. Individual segments were detrended and baseline corrected over the 4096 ms interval. Segments were considered artifacts and rejected from analysis if activity in any scalp or EOG channel exceeded ±80 μV. Segmented data were then time averaged separately for each condition of load and ring eccentricity. Frequency-domain signals (8.3 Hz) were extracted by submitting time-averaged data to FFT (10% Hanning window). 8.3 Hz amplitudes were submitted to 3 × 2 × 3 repeated measures ANOVA with factors of electrode (O1, Oz, or O2), load (low load or high load), and eccentricity (2, 6, or 11° rings).

#### ERPs

Time-domain ERPs to task-relevant stimuli at fixation were calculated by averaging 700 ms segments of EEG time-locked to each stimulus presentation. Individual segments were rejected from analysis if activity exceeded ±80 μV. Segmented data were averaged separately for low load and high load conditions. Visual sensory components, P1 and N1, were selected a priori for statistical analysis. Mean amplitudes within 110–150 and 175–210 ms time windows were used to quantify P1 and N1 amplitudes, respectively. P1 and N1 amplitudes were submitted to separate repeated measures ANOVAs with factors of electrode (P7 or P8), load (low or high load), and distractor type (2, 6, or 11°, or no distractor).

## Results

### Behavioral performance

Consistent with the increased attentional demands of high attentional load blocks, responses to target stimuli appearing at fixation were faster and more accurate under low load than high load. Paired samples *t*-tests were used to test behavioral differences between low and high load performance. Reaction times were faster under low attentional load, *t*_(19)_ = −23.59, *p* < 0.001 (low load: *M* = 371 ms, *SD* = 41 ms; high load: *M* = 526 ms, *SD* = 52 ms). Subjects were also more accurate under low load, *t*_(19)_ = 4.03, *p* < 0.001 (low load: *M* = 0.993, *SD* = 0.020; high load: *M* = 0.970, *SD* = 0.041).

### Frequency-domain SSVEPs

**Figure 2:**
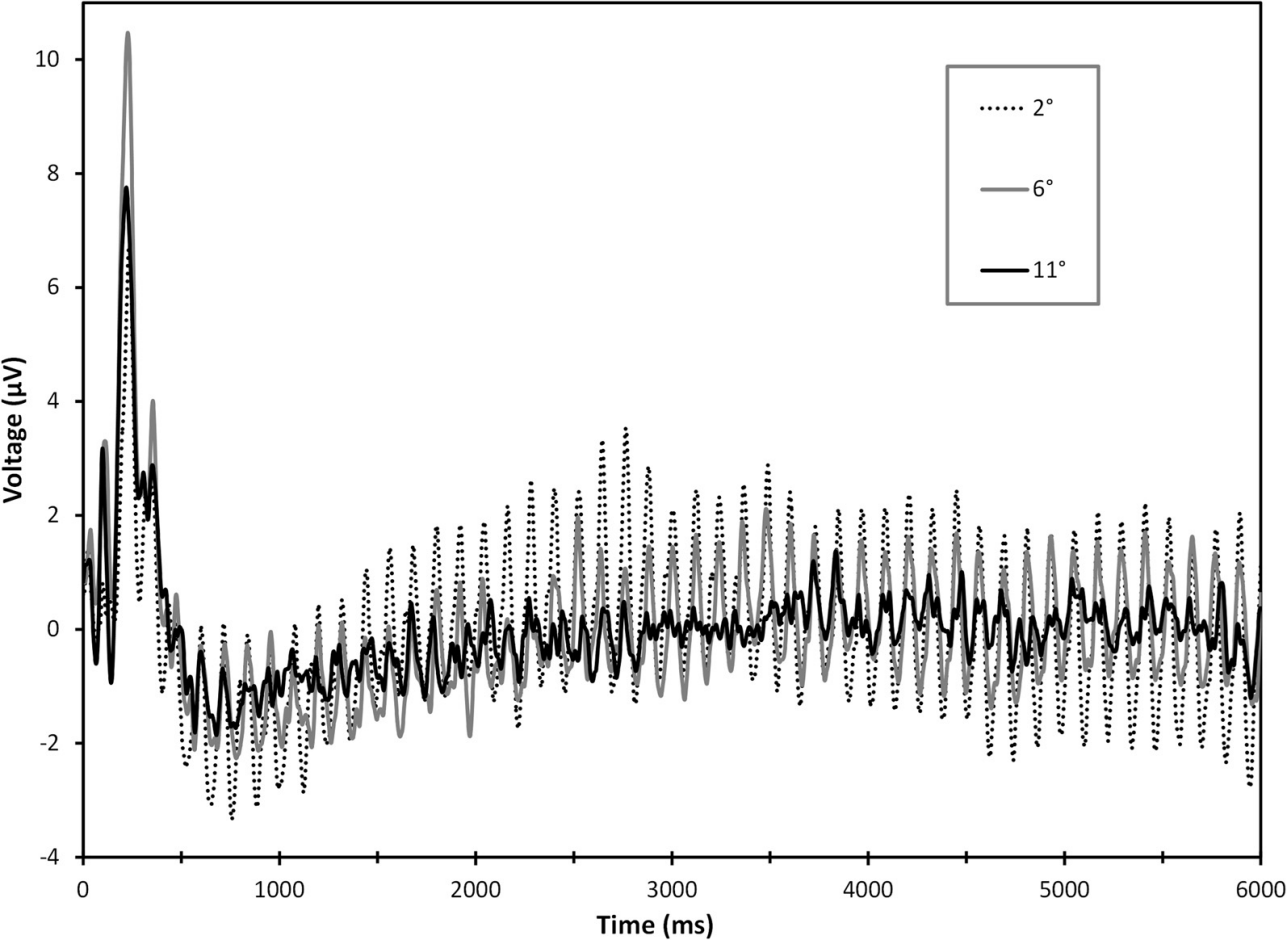
**Grand average time-domain responses for 8.3 Hz SSVEP responses over the course of a 6.0 s trial, collapsed across electrodes O1, Oz, and O2.** Notice a large initial transient visual response to the onset of peripheral ring distractors followed by entrainment of the steady-state response. Frequency-domain signals of SSVEPs were extracted from a period of 4096 ms beginning 1500 ms into the trial.

**Figure 3:**
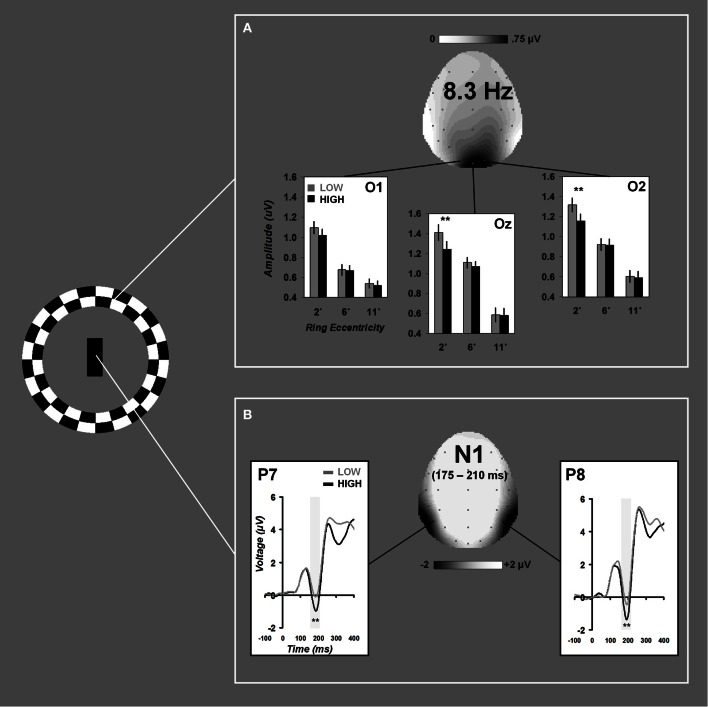
**Load-dependent effects on distractor and task-relevant visual processing.** Grand average effects of attentional load on 8.3 Hz peripheral distractor amplitudes are plotted for electrodes O1, Oz, and O2 in **(A)**. Grand average time-domain ERPs to foveal task-relevant stimuli at electrodes P7 and P8 are shown in **(B)**. Scalp distributions reflect the grand averages collapsed across all conditions. Error bars represent ±1 SEM.

A significant electrode × load × eccentricity interaction was found for 8.3 Hz steady-state amplitudes, *F*_(4, 76)_ = 2.93, ε = 0.708, *p* = 0.045. Follow-up ANOVAs were performed at each electrode and revealed significant load × eccentricity interactions at Oz, *F*_(2, 38)_ = 4.01, ε = 0.675, *p* = 0.045, and O2, *F*_(2, 38)_ = 4.99, ε = 0.722, *p* = 0.023. Paired-samples *t*-tests comparing differences between low and high load at each eccentricity revealed that distractor SSVEPs were attenuated under high load at the most proximal eccentricity (2°), Oz: *t*_(19)_ = 3.09, *p* = 0.006, and O2: *t*_(19)_ = 3.51, *p* = 0.002. No differences due to load were present for rings at 6 or 11° eccentricities (all p's >0.28). Grand average time-domain visual responses from SSVEP trials are plotted for each eccentricity in Figure 2. Grand average scalp distribution and load-dependent SSVEP amplitudes for each eccentricity are plotted in Figure 3A.

### Time-domain ERPs

Analysis of P1 amplitudes revealed a main effect of distractor type, *F*_(3.57)_ = 10.61, ε = 0.863, *p* < 0.001, but no significant effects of attentional load (all *p*'s > 0.07). The main effect of distractor type resulted from increased P1 amplitude with increasing eccentricity of peripheral distractors (or their absence), supported by mean P1 amplitudes across conditions of distractor type (2°: *M* = 1.31 μV, *SD* = 1.00 μV; 6°: *M* = 1.59 μV, *SD* = 1.12 μV; 11°: *M* = 1.73 μV, *SD* = 1.26 μV; no distractor: *M* = 2.15 μV, *SD* = 1.40 μV) and a significant linear trend of distractor type, *F*_(1, 19)_ = 30.17, *p* < 0.001. This relationship between P1 suppression and distractor proximity may reflect visual competition between task-relevant foveal stimuli and peripheral distractors, as described previously by Parks et al. (2011). However, this result will not be interpreted further as investigating such an effect was not a goal of the present experiment nor was there interaction with attentional load.

The N1 ANOVA revealed a significant main effect of load, *F*_(1, 19)_ = 45.94, *p* < 0.001 resulting from greater N1 amplitude under high attentional load relative to low. Grand average time-domain ERPs and scalp distribution are plotted in Figure 3B.

## Discussion

The perceptual load theory of attention proposes that irrelevant or distracting stimuli become filtered as the attentional demands of a task increase (Lavie and Tsal, 1994; Lavie, 1995). Though the theory does not specify a neural mechanism of these effects, Torralbo and Beck (2008) have proposed that such perceptual load is a consequence of a top–down biasing signal being driven by neural competition. This proposal, in conjunction with the predictions of biased competition and normalization theories of attentions, predicts that stimulus locations rendered high in perceptual load will result in a neural enhancement of the attentionally demanding stimulus while simultaneously inducing a region of visual suppression in spatially proximal locations. We used time-domain ERPs together with frequency-domain SSVEPs to test this proposition by examining load-dependent enhancement and suppression in the visual field.

Time-domain ERPs elicited by foveal task-relevant stimuli revealed evidence of enhanced visual processing under high perceptual load. Specifically, amplitude of the posterior N1 component was significantly potentiated in the high load condition relative to low. This finding is consistent with several previous ERP studies which have reported increased N1 amplitude to high load task-relevant stimuli (Handy and Mangun, 2000; Rorden et al., 2008; Rauss et al., 2009, 2012). The visual posterior N1 has been proposed to reflect processes of stimulus discrimination, as it has been found to increase in amplitude when subject must discriminate stimuli relative to when no discrimination is required (Vogel and Luck, 2000). The N1 modulation observed in our experiment and others (Handy and Mangun, 2000; Rorden et al., 2008; Rauss et al., 2009, 2012) may be related to such a process but our results clearly demonstrate that N1 amplitude modulates with the attentional load of a task. Vogel and Luck (2000) previously tested whether perceptual load could account for discrimination effects in the N1 and found no effect of load. However, their manipulation of perceptual load varied the similarity of color between targets and distractors to influence task difficulty. Such a manipulation may have increased task difficulty through sensory limitations rather than attentional demands, which have been shown to be distinctly different methods of manipulating task difficulty (Lavie and De Fockert, 2003).

A potential alternative interpretation of the load-dependent N1 modulation reported here is that it result from differential attentional capture between high and low load conditions (Folk et al., 1992; Fuchs and Ansorge, 2012; Fuchs et al., 2013). In the high load condition, every no-go stimulus matched the color of one of the assigned targets whereas no-go stimuli in low load never matched the color of assigned targets. As such, no-go stimuli in the high load condition may have induced attentional capture by color whereas no-go stimuli in the low load condition may not induce such capture. This imbalance of attentional capture between low and high load conditions could potentially have led to a load-dependent modulation of the N1 modulation. Such an interpretation cannot be ruled out in the present experiment as the increased attentional demands of the high load condition predict an effect in the same direction (i.e., modulation of visual sensory components). However, previous studies of attentional load have also described such an N1 effect but have used manipulations that do not differentially influence attentional capture (Rorden et al., 2008; Rauss et al., 2009, 2012). We propose that the increased N1 reported here reflects an enhancement in perceptual processing occurring as a result of the increased attentional demands required under high perceptual load, and potentially mediated by a top–down biasing signal.

Evidence of the suppression of visual distractor stimuli was apparent in 8.3 Hz distractor SSVEPs. Parametric manipulation of distractor eccentricity revealed that high central load attenuated 8.3 Hz distractor signals at the most proximal position (2° from the task-relevant location). No evidence of load-dependent distractor suppression was apparent at eccentricities beyond 2° (6 or 11°). These findings are consistent with the predictions of surround suppression and biased competition theory (Reynolds et al., 1999) and normalization theory (Reynolds and Heeger, 2009), and demonstrate that increased attentional load at fixation induces a relatively narrow region of distractor suppression surrounding the spatial location with increased attentional demand, rather than inducing uniform filtering of distractor stimuli throughout the visual field.

Our SSVEP results showed the strongest suppression at the spatial position nearby the attentionally demanding central load task but did not reveal significant suppression at further distances. However, previous psychophysical and neuroimaging data have shown evidence of load-dependent visual suppression at eccentricities beyond 10°. Plainis et al. (2001) previously reported increased visual detection thresholds in a foveal load task for spatial locations up to 10° from fixation but significantly less suppression for eccentricities beyond 10°. However, these results were based on behavioral responses rather than neurophysiological recordings and the use of a secondary psychophysical task for threshold measurement was likely to have influenced subjects' attentional strategies and, in turn, the measured spatial configuration of distractor suppression. Some neurophysiological data have also indicated that load-induced visual suppression is measurable at distant eccentricities. A previous fMRI study by Schwartz et al. (2005) found some load-dependent suppression within retinotopically-organized cortex presumed to represent eccentricities beyond those reported here (>2°). However, in their fMRI study, distractor stimuli were near full-hemifield checkerboard wedges. Object-based and space-based mechanisms of attention have been shown to interact such that spatial effects can “spread” within an object's spatial boundaries and retinotopic representations (Vecera and Farah, 1994; Kramer et al., 1997; Müller and Kleinschmidt, 2003). If the large distractor stimulus used by Schwartz et al. was grouped as an object, it is possible that distractor suppression spread from less eccentric to more eccentric representations. Furthermore, Schwartz et al. extracted eccentricity information from fMRI activations obtained in retinotopic mapping scans of visual cortical areas, which did not discretize comparisons of load effects across the visual field. The present study placed discrete objects (checkerboard rings) at known eccentricities, minimizing any potential space-object interactions and avoiding interpretation issues associated with retinotopic extraction of cortical representations of eccentricity. Though our parametric manipulation of eccentricity provides a straightforward method of examining load-dependent modulations across visual space, it could be argued that overall differences in SSVEP amplitude between 2, 6, and 11° distractors negatively impacted our ability to detect attentional effects, as SSVEP signals progressively decreased in amplitude from 2, 6, and 11°, despite scaling ring size for cortical magnification (Figures 2, 3A). Although we cannot completely rule out that we simply were unable to measure more peripheral effects of attention, we do not believe the SSVEP amplitude differences to be of major concern as all eccentricities exhibited a robust 8.3 Hz signal and even the most eccentric position in our experiment (11°) exhibited an average frequency-domain amplitude greater than 0.5 μV, a value comparable to signals of previous studies of spatial attention using SSVEPs (Müller et al., 1998a,b, 2003; Müller and Hübner, 2002). Though the existence of a diminutive effect of perceptual load at more peripheral locations remains a possibility, our SSVEP data clearly indicate that load-dependent visual suppression has the most pronounced effects in the regions of visual space directly surrounding task-relevant stimuli.

Together, results from time-domain ERPs and frequency-domain SSVEPs indicate that attentional load induces a center-surround configuration of facilitation and suppression in the visual field. Specifically, enhancement of perceptual-level processing was present at the central task-relevant location (load-dependent N1 effect) whereas suppression was apparent only in a region of space surrounding this location (2° load-dependent SSVEP effect). Such a configuration is in accordance with predictions from normalization theory of attention, with the simple assumption that the “suppressive drive” of a neuron is spatially restricted at least in early to intermediate levels of visual cortex (Reynolds and Heeger, 2009) and is further consistent with previously reported findings of a center-surround distribution of *spatial* selective attention to peripheral locations (Müller and Kleinschmidt, 2004; Müller et al., 2005; Hopf et al., 2006; Boehler et al., 2009). Though the present results demonstrate a center-surround distribution they provide a relatively crude resolution of measurement, as load-dependent comparisons were made 2, 6, and 11° from fixation using stimuli scaled for cortical magnification. It is possible that taking finer-resolution measurements between 2 and 6° could reveal a more complex configuration of facilitation and suppression. Furthermore, it remains unclear whether the present results reflect a static configuration where suppression always occurs at predetermined regions of the visual field, or a dynamic center-surround configuration that scales according to the attended region of space (e.g., Reynolds and Heeger, 2009). For example, if the task-relevant location encompassed an area 3° in diameter, the relative distribution of facilitation and suppression may be predicted to scale accordingly. The aforementioned possibilities can only be addressed with, potentially through further experimentation with an adapted version of the paradigm used here.

A recent set of studies has called into question the validity of perceptual load theory [Benoni and Tsal, 2010, 2012; Tsal and Benoni, 2010a,b however, see Lavie and Torralbo (2010)] and should be discussed in the context of the current experiment. Dilution theory purports that the described effects of perceptual load in search displays are not due to increased attentional distractor suppression but are due to the dilution of distractor items by a large number of neutral items. The present study used identical stimuli between low and high load conditions and manipulated attentional demands through the task performed on those stimuli (i.e., targets were assigned by color or by color/orientation). As there were no stimulus differences between the configuration of low load and high load displays, dilution cannot account for our findings of central visual enhancement and suppression by load. The intention of our study was not to directly compare perceptual load and dilution theories. However, the load-dependent ERP and SSVEP effects described here are clearly the result of the attentional demands induced by the central load task rather than an effect of dilution by distractor items.

In summary, our results demonstrate that load-dependent distractor filtering assumes a center-surround configuration. Time-domain ERPs and frequency-domain SSVEPs revealed that, under conditions of high perceptual load, visual processing is enhanced at a task-relevant location but is suppressed in the space immediately surrounding that location.

### Conflict of interest statement

The authors declare that the research was conducted in the absence of any commercial or financial relationships that could be construed as a potential conflict of interest.
